# High-Throughput Continuous Free-Flow Dielectrophoretic Trapping of Micron-Scale Particles and Cells in Paper Using Localized Nonuniform Pore-Scale-Generated Paper-Based Electric Field Gradients

**DOI:** 10.1021/acs.analchem.3c03740

**Published:** 2024-01-09

**Authors:** Md. Nazibul Islam, Bhavya Jaiswal, Zachary R. Gagnon

**Affiliations:** Artie McFerrin Department of Chemical Engineering, Texas A&M University, College Station, Texas 77843, United States

## Abstract

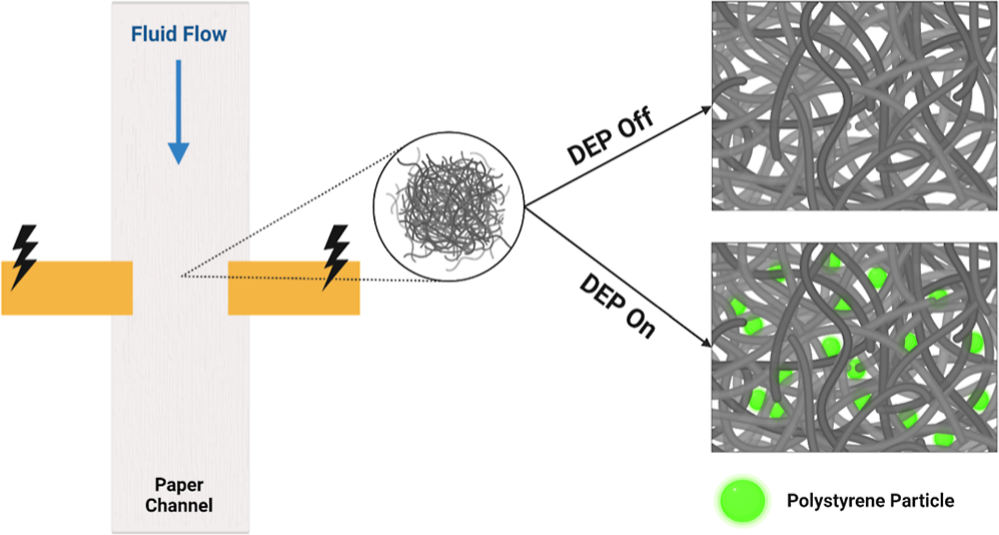

Dielectrophoresis (DEP) utilizes a spatially varying nonuniform electrical field to induce forces on suspended polarizable soft matter including particles and cells. Such nonuniformities are conventionally created using 2D or 3D micrometer-scale electrode arrays. Alternatively, insulator-based dielectrophoresis (iDEP) uses small micrometer-scale insulating structures to spatially distort and generate regions of localized field gradients to selectively trap, isolate, and concentrate bioparticles, including bacteria, viruses, red blood cells, and cancer cells from a suspending electrolyte solution. Despite significant advances in the microfabrication technology, the commercial adoption of DEP devices for soft matter manipulation remains elusive. One reason for low market penetration is a lack of low-cost and scalable fabrication methods to quickly microfabricate field-deforming structures to generate localized DEP-inducing electric field gradients. We propose here that paper-based devices can offer a low-cost and easy-to-use alternative to traditional iDEP devices. In this article, we demonstrate for the first time the ability to perform iDEP-style particle trapping using the naturally occurring micrometer-scale insulating porous structures of paper. In particular, we use polymeric laminated nonwoven fiberglass paper channels as a source of insulating structures for iDEP. We apply a flow of polarizable microparticles directly within the nonwoven channel and simultaneously drop an electric field perpendicular to the flow direction to induce DEP. We show the ability to readily trap and concentrate particles in paper by DEP with an applied voltage as low as 2 V using two different flow mechanisms: a constant fluid flow rate using an external pump and passive fluid flow by capillary wicking. Using a combination of micro computed tomography and finite element analysis, we then present a computational model to probe the microscale DEP force formation dynamics within the paper structure. This new paper-based iDEP platform enables the development of robust, low-cost, and portable next-generation iDEP systems for a wide variety of sample purification and liquid handling applications.

## 1 Introduction

Insulator-based dielectrophoresis (iDEP) has emerged as an electrokinetic tool for manipulation of biomolecules in micro total analysis systems and lab-on-a-chip (LOC) devices. The iDEP method, as the name implies, utilizes micrometer-scale insulating structures to induce curvature in the electric field lines which pass across these objects. Such curvature induces localized regions of electric field gradients and a subsequent dielectrophoretic force. These forces can be used to trap and remove suspending particles, cells, and biomolecules from the surrounding flow field. iDEP is typically biologically compatible and can maintain mammalian cell viability for up to an hour, whereas more traditional electrode-based dielectrophoresis (DEP) devices can impact cell viability within milliseconds.^1^ In addition, iDEP has been used in a wide range of other LOC applications, including biomarker screening, exosome trapping, manipulation of viruses, protein folding analysis, and molecular sensing.^1−5^

The first report of iDEP was published in 1989, where Masuda et al. utilized insulating pillar structures to trap cells, and subsequently a pulsed voltage, to initiate cellular fusion.^6^ Another breakthrough was published in 2000 by Singh et al., who used an array of glass-etched insulating posts to trap flowing particles using a combination of linear electro-osmotic and DEP forces.^7^ In 2002, Chou et al. coined the term “electrodeless dielectrophoresis” and trapped single- and double-stranded DNA.^8^ In general, iDEP is well-defined experimentally; researchers have studied the effects of media conductivity, pH, and electrode geometry on particle trapping^9,10^ and demonstrated iDEP-based concentration and separation of bacteria and yeast cells,^11,12^ human red blood cells,^12^ and ovarian cancer and prostate tumor-initiating cells.^13−16^ Others have creatively varied iDEP insulator shape and geometry developing, for example, devices with converging “sawtooth” geometries using triangular teeth-like structures.^17^ Such sawtooth structures can manipulate bacteria, viruses, Aβ amyloid fibrils, human blood cells, and molecular biomarkers.^18−21^ Furthermore, iDEP can be used to trap free-flowing particles, proteins, and nucleic acids within a flow field as they flow across insulating structures.^22−24^

One disadvantage of iDEP is that the insulating structures required for DEP force generation can require tedious and expensive microfabrication/cleanroom techniques. One way to overcome this disadvantage is to utilize naturally occurring porous materials, like alumina, to emulate insulating pillars and spatially manipulate the electric field.^25−27^ These novel pore-scale devices use the insulating porous microstructure to naturally generate regions of localized high electric field strength and have been used to successfully separate polystyrene (PS) particles and bioparticles (*Saccharomyces cerevisiae*, yeast) at flow rates several orders of magnitude greater than typical microfluidic-based iDEP devices.^25−27^ However, despite such academic advances, commercial adoption of iDEP remains low as insulating alumina is not readily available commercially and requires significant chemistry expertise to synthesize.

One reason for limited adoption of iDEP, in general, is a lack of low-cost manufacturing methods capable of scalably producing fluidic chips with micrometer-scale insulating structures capable of inducing localized DEP forces. While commercial techniques such as injection molding can produce such features, they often require a six-figure upfront investment for both development and production phases of commercialization. Such high costs can constrain and severely limit academic translation and widespread consumer adoption. Therefore, low-cost iDEP devices capable of being scalably manufactured from low-cost and readily available materials will have a significant impact in democratizing innovation and commercialization of this powerful electrokinetic method.

Over the past decade, paper-based microfluidics has received significant interest for use in ultralow-cost diagnostics.^28^ Conventionally powered passively by liquid wicking, paper-based devices have been extensively developed for lateral flow assays and colorimetric detection devices.^29−31^ In addition, several groups have integrated electrokinetic-based phenomena, including electrophoresis and isotachophoresis, with paper.^32−35^ In 2019, for example, Chakraborty et al. reported simple particle manipulation on paper using conventional electrode-based DEP.^32,36,37^ In this device, an electric field was applied across a small paper-electrode gap using two pencil-drawn graphite electrodes to maneuver PS particles across cellulose filter paper by DEP.^37^ Particles were observed to move from zones of high electric field to low electric field, as defined by the electrodes themselves but not by the paper geometry or the underlying porous structure. In addition, they demonstrated concentration of *Escherichia coli* bacteria near the pencil-drawn positively polarized electrode.^37^ While these studies demonstrate that paper is capable of transmitting an electrical field for DEP, none take advantage of the naturally occurring porous and fibrous structures. In particular, given the availability of paper substrates with insulating fibers, we hypothesized that insulating pore-scale structures can serve to emulate conventional pillar-style arrays typically used in iDEP applications. The investigation of this hypothesis is the purpose of this study.

In this work, we report a novel iDEP technique that employs insulating paper fibers as micrometer-scale electric field-deforming structures. This “paper-DEP” method uses such structures to generate localized iDEP forces within low-cost porous paper substrates for soft matter manipulation applications. We use a synthetic fiber paper, typically described as a “nonwoven”, comprised of a compressed network of electrical grade glass fibers to serve as DEP field gradient-inducing electrical insulators.^38,39^ Soft matter suspensions are driven into these porous substrates using the microfluidic technique, microfluidic pressure in paper (μPiP), which enables encapsulated paper fluidic channels to sustain continuous pressure-driven fluid flow or a wicking-based flow without evaporation. We integrate a pair of conductive electrodes across the microchannel width in a direction perpendicular to the fluid flow to generate pore-scale local electric field gradients to drive iDEP-based particle and cellular trapping within the paper pores.

We first characterize the iDEP trapping behavior within paper and quantitatively demonstrate the existence of pore-scale electric field gradients using a combination of microcomputed tomography (micro-CT) and finite element analysis (FEA). We then demonstrate iDEP particle trapping under varying electric fields and flow conditions. Finally, we conclude our investigation by demonstrating paper DEP biocompatibility by trapping fluorescently labeled *E. coli* bacteria. To the best of our knowledge, this marks the inaugural use of paper fibers as insulating field gradient-generating structures for inducing iDEP-style trapping within paper pores. This platform offers a cost-effective, microfabrication-free method for iDEP-based applications, catering to both academic prototyping and large-scale commercial device production.

## 2 Theory

When an electric potential is applied across a porous paper channel, the resulting electric field is compelled to converge and diverge across the pore spaces due to geometric constraints. Here, fibers act as insulating structures and serve to induce electric field line curvature and an electric field gradient around each insulating fiber. The DEP force acting on a spherical particle moving through the paper structure is dependent on this electric gradient and can be expressed as^40,41^
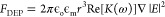
1where, ϵ_o_, ϵ_m_, *r*, and ∇|*E*|^2^ are the permittivity of free space, dielectric constant of the media, particle radius, and the square of the electric field gradient, respectively. The sign of the DEP force is dependent on the real part of the Clausius–Mossotti factor (CMF), Re[*K*(ω)]^40,41^
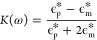
2

The CMF is a complex term with both real and imaginary parts, serving to describe the polarizability of an electroneutral particle suspended in an electrolyte medium. The expression for ϵ_i_^*^ is given by ϵ_i_^*^ = ϵ_i_ – *i*σ/ω, where ϵ_i_ is the dielectric constant of the particle (*p*) or medium (*m*), σ is the electrical conductivity of the medium, and ω is the electric field frequency.^41,42^ The DEP force is contingent on the real part of *K*(ω), which represents the component of the induced dipole moment in phase with the electric field.^42^ The electric field itself is determined by computing the negative gradient of the electric potential (ϕ): *E* = −∇ϕ. For electroneutral particles and media, this computation satisfies the Laplace equation: ∇^2^ϕ = 0. In addition to the DEP force, polarized particles flowing through a viscous fluid-filled paper pore at a nonzero velocity experience a drag force, *F*_D_, as described by Stokes’ law.^43−45^ Within a paper pore, the quantitative threshold for DEP particle trapping is met when the DEP force equals or surpasses the drag force.^44,45^ Consequently, a trapping zone within the porous structure is established when localized pore-scale DEP forces exceed the particle drag force exerted by the moving fluid.

## 3 Methods

### 3.1 Device Fabrication

**Figure 1:**
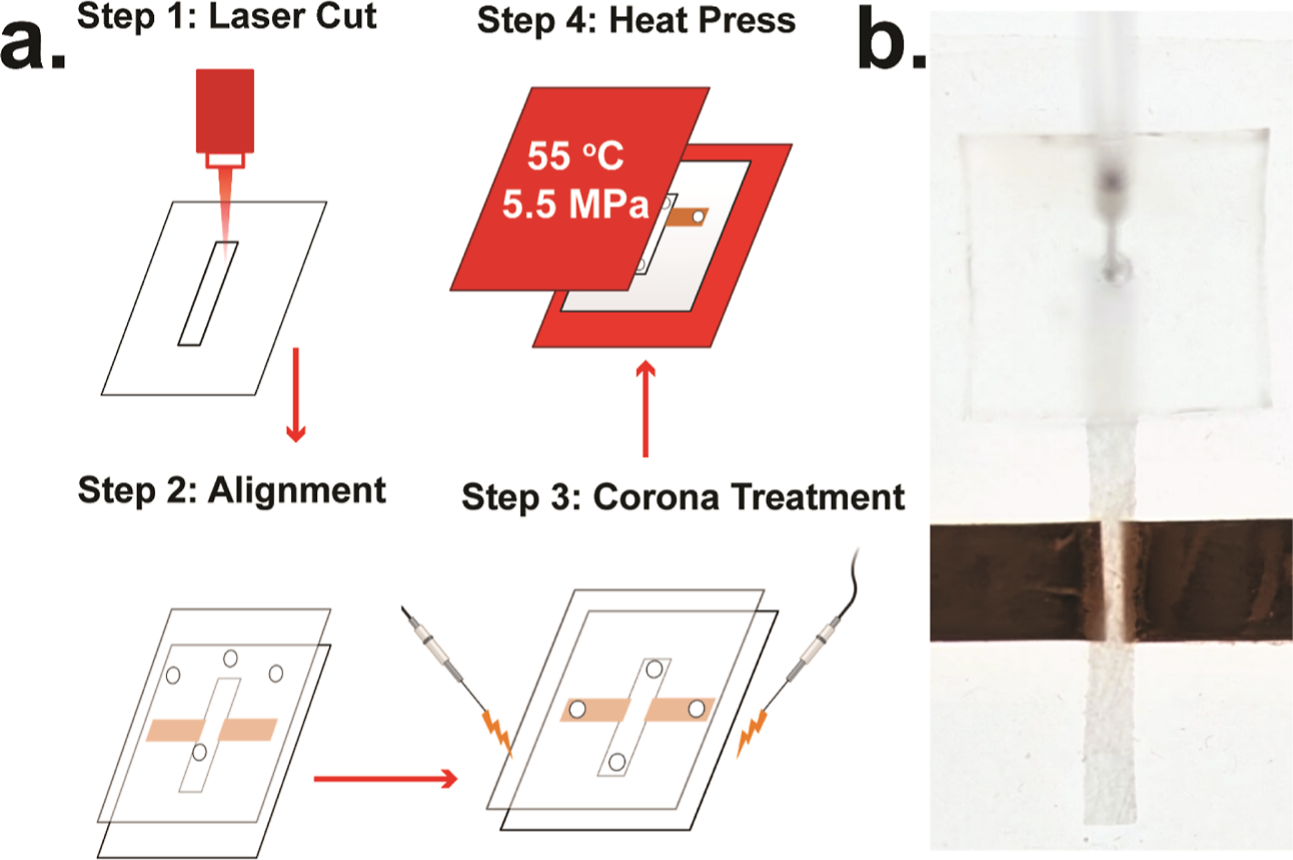
A) Fabrication workflow for a paper-based dielectrophoretic trapping device. (B) Fully assembled and sealed paper-based DEP device with integrated copper electrodes.

**Figure 2:**
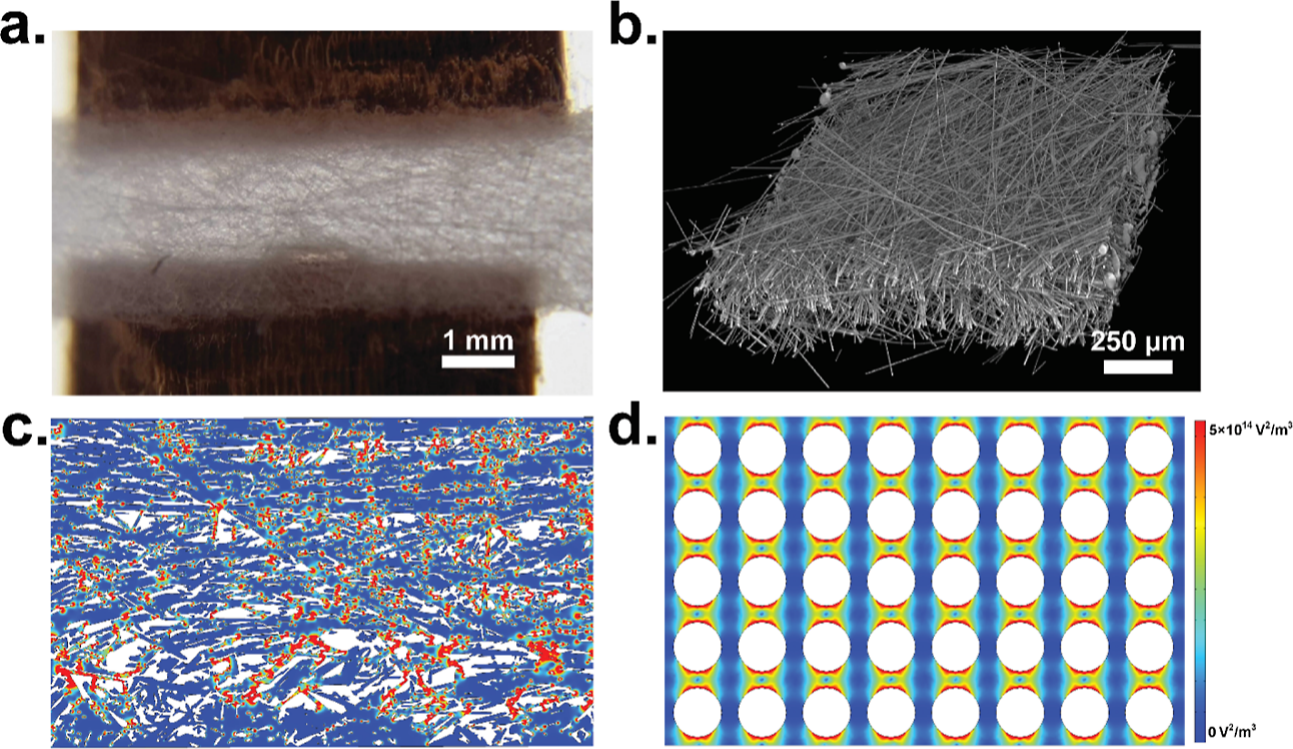
Micro-CT imaging and FEA analysis. (A) Paper-based DEP device. (B) Micro-CT scan of a paper substrate. (C) 2D electric field gradient distribution demonstrates localized DEP forces. (D) Paper-based gradients are similar in nature to a traditional iDEP device.

The entire fabrication process, from design to device, can be completed in less than 10 min. Paper devices were manufactured using the μPiP fabrication process, as depicted in Figure 1.^28^ First, a straight channel geometry (4 mm × 40 mm) was cut from a sheet of Craneglas 230 paper (Neenah Filtration) by using a CO_2_ laser cutter (LS-2440, Boss Laser). A pair of copper tape electrodes (9 mm × 30 mm, 3 M Copper Foil) were precisely positioned along the edges of the channel, leaving a 1 mm gap across the paper channel width. The paper channel and copper tape electrodes were sandwiched between two polydimethylsiloxane (PDMS) sheets (0.5 mm, McMaster-Carr), as depicted in Figure 1b. To generate a minimum fluidic resistance, the fluidic flow direction was aligned along the preferential micro-CT-imaged fiber direction. Fluidic channel inlets and outlets were punched on the top PDMS sheet using a 0.75 mm biopsy punch (Ted Pella, Inc.). Sheets were oxidized and bonded using oxygen plasma (Electro-Technic Products, Model BD-20AC), thermally compressed using a benchtop heat press (Dulytek DW 400) for 5 min at 55 °C, and copper wires were pierced into each electrode via the top PDMS sheet for connection to an external voltage source (Figure 1c). Finally, to minimize unwanted particle adsorption, sealed paper channels were soaked in 3% (w/v) BSA (Millipore-Sigma) in deionized water (DI) for 20 min and dried prior to use. A brightfield micrograph of the assembled device is shown in Figure 2a.

### 3.2 Micro-CT Analysis of Paper Channels

The paper substrate was quantitatively imaged using micro-CT to investigate the porous structure of the insulating fibers and to compute the substrate’s electric field and DEP force distribution. Scans were performed with the SkyScan 1272 benchtop micro-CT scan (Micro Photonics Inc.; 1.5 μm voxel size; 800 ms exposure time). Scans were digitized using CTvox software and then imported into a finite element Multiphysics package (COMSOL Inc., Burlington, MA) for FEA. Laplace’s equation was solved by treating paper fibers as insulating no-flux boundaries surrounded by an aqueous water domain. Each computational model consisted of a 2D slice with an element mesh consisting of 810,343 triangular and 142,400 quadrilateral mesh elements.

### 3.3 Experimental Setup

Fluorescent PS microparticles (1 μm diameter, Bangs Laboratories, Inc.) were suspended in DI and diluted to a concentration of 8 × 10^5^ particles per mL (conductivity: 2 μS/cm, pH: 6.8), 5 wt % Tween 20 was added to the solution to reduce particle agglomeration. *E. coli* DH5α cells (Thermo Fisher Scientific, Waltham, USA) were cultured overnight at 37 °C and 250 rpm in a 15 mL falcon tube containing Luria broth medium. For experimentation, *E. coli* cells were washed with DI twice at 3000 rpm for 5 min, resuspended in DI, and diluted to a concentration of 2.16 × 10^7^ CFU. SYBR Green-I (SYBR) was used to stain bacterial nucleic acids for confocal imaging. Devices were imaged fluorescently under swept field confocal microscopy (Nikon Eclipse Ti), 70 μm confocal slit (Nikon/Prairie Technologies), an Andor iXon 897 EMCCD camera, and a 20× objective. Fluorescent intensity (*I*) measurements were performed using ImageJ, 1.47t and plotted normalized by the intensity at the baseline voltage as *I*/*I*_*i*V_, where *i* is a baseline voltage (0 or 2 V) for a given experiment.

## 4 Results and Discussion

### 4.1 FEA

We first captured micro-CT images of the paper substrate to quantitatively analyze its 3D structure (Figure 2b). As shown in Figure 2b, the paper fiber orientation was observed to be anisotropic, where one axial fiber orientation is aligned to a greater degree than all of the other axes. We imported a 2D slice of the 3D digitized geometry into a FEA package and computed the microscale electric field gradients within the paper pores. Such a 2D approximation has been successfully used to model iDEP electric fields based on the assumption that the electrical potential is approximately constant across the small depth of the fluidic channel.^21,47,48^ We computed the gradient of the electric field squared (∇|*E*|^2^) for both the paper structure (Figure 2c) and a traditional iDEP device (Figure 2d). As shown in Figure 2c, localized field gradient zones are distributed unevenly across the domain compared to a conventional iDEP device. However, it is clear that the insulating structure produces localized pore-scale regions of electric field gradient, similar to that of the traditional iDEP device. It is worth noting that electrophoresis of the second kind was not considered for this analysis and only zones of high electric field gradient are considered as trapping zones. Electrophoresis of the second kind has been shown to be generated at much greater field strengths (∼500 V/cm) than that used in this work.^4,46^

**Figure 3:**
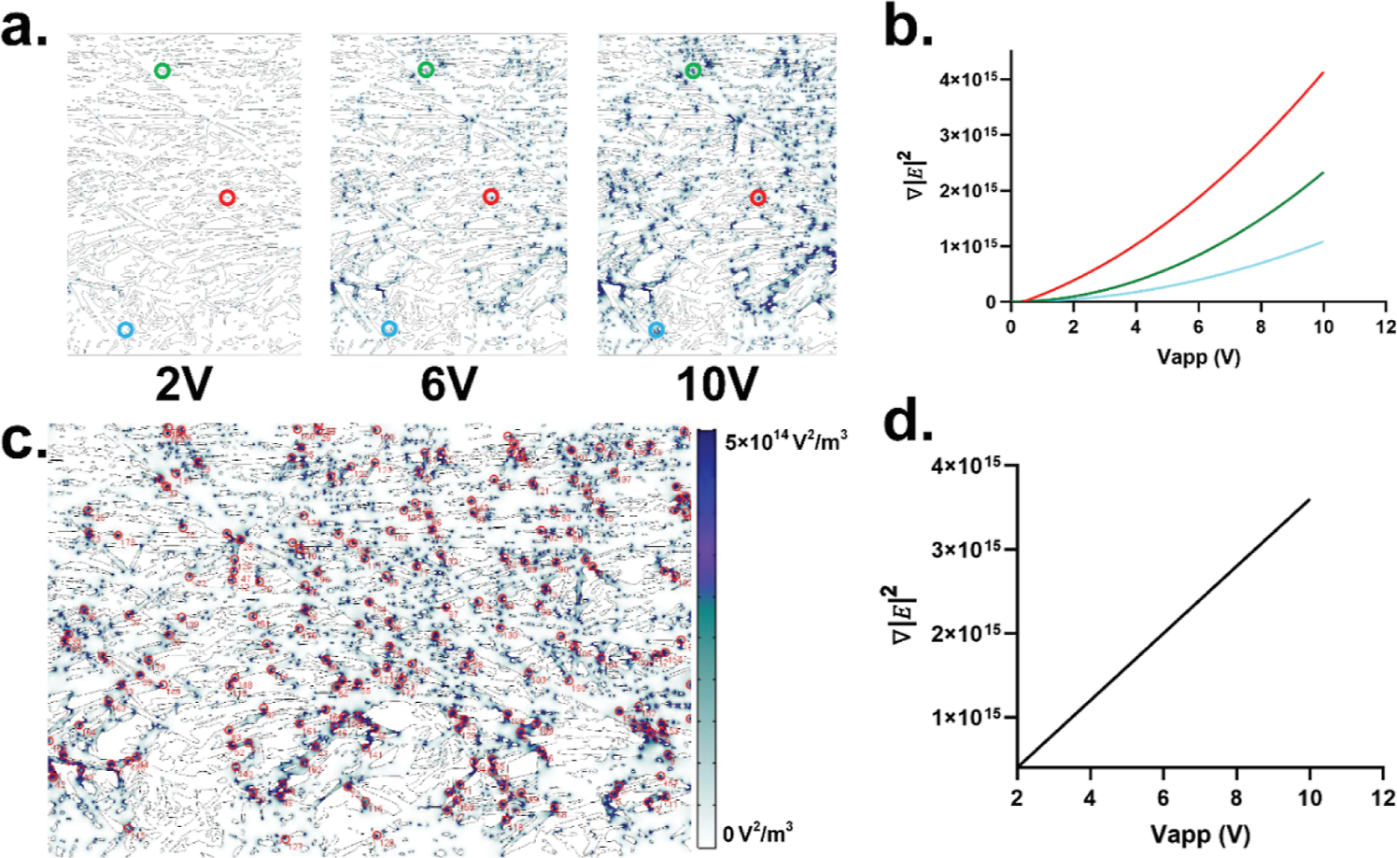
DEP force growth dynamics and trapping mechanism within the paper pores. (A) DEP force at different applied voltages within paper pores. (B) DEP force growth curves for three different trapping zones. (C) DEP trapping zones within paper pore. (D) Spatially averaged DEP force growth curve shows linear growth dynamics.

To better understand the DEP force growth dynamics, we quantified ∇|*E*|^2^ at varying voltages at three spatial DEP trapping zone locations, as shown in Figure 3a. Here, a trapping zone is defined as pore-scale locations within the paper structure with a high local DEP force capable of trapping a moving particle from the flow field. As shown in Figure 3b, both the number of trapping zones and the trapping volume, which is the local volume where trapping occurs, increased with the applied voltage (Vapp). However, while the DEP force in each trapping zone was observed to grow quadratically with applied voltage, each localized force growth rate is not equivalent. We observed that the DEP force in trapping zones with sharp fiber curvature grew at a faster rate than trapping zones with more rounded fiber orientations. To understand the localized-bulk trapping growth dynamics, we computed the spatially averaged DEP force growth curve captured from 194 randomly selected trapping zones (Figure 3c) and plotted this average against the applied voltage (Figure 3d). Interestingly, the resulting averaged force growth curve shows a linear relationship with the applied voltage over 0–10 Vapp (Figure 3d).

### 4.2 AC DEP in Paper

Our FEA model suggests that insulating fibrous paper can generate pore-scale DEP trapping zones. We next performed “classical” DEP experiments with paper using an AC electric field over varying field frequencies (100 kHz–5 MHz) and compared DEP assembly dynamics with a conventional 2D quadrupole electrode array. Both paper and electrode DEP experiments were performed with fluorescently labeled PS particles suspended in DI. For paper DEP, PS particles were flowed through the paper channel at a constant flow rate of 4 μL/min. The quadrupolar electrode device utilized a 5 μL PS suspension pipetted directly atop the electrode array. A function generator (Rigol DG4102) delivered a 20 V peak-to-peak sinusoidal voltage to every other quadrupole electrode, while the other two electrodes were grounded. For paper DEP, the function generator was connected to a 40 dB gain RF amplifier (Mini-Circuits, ZHL-5W-1+) to deliver an oscilloscope-measured 6.2 V peak-to-peak voltage across the copper electrodes.

**Figure 4:**
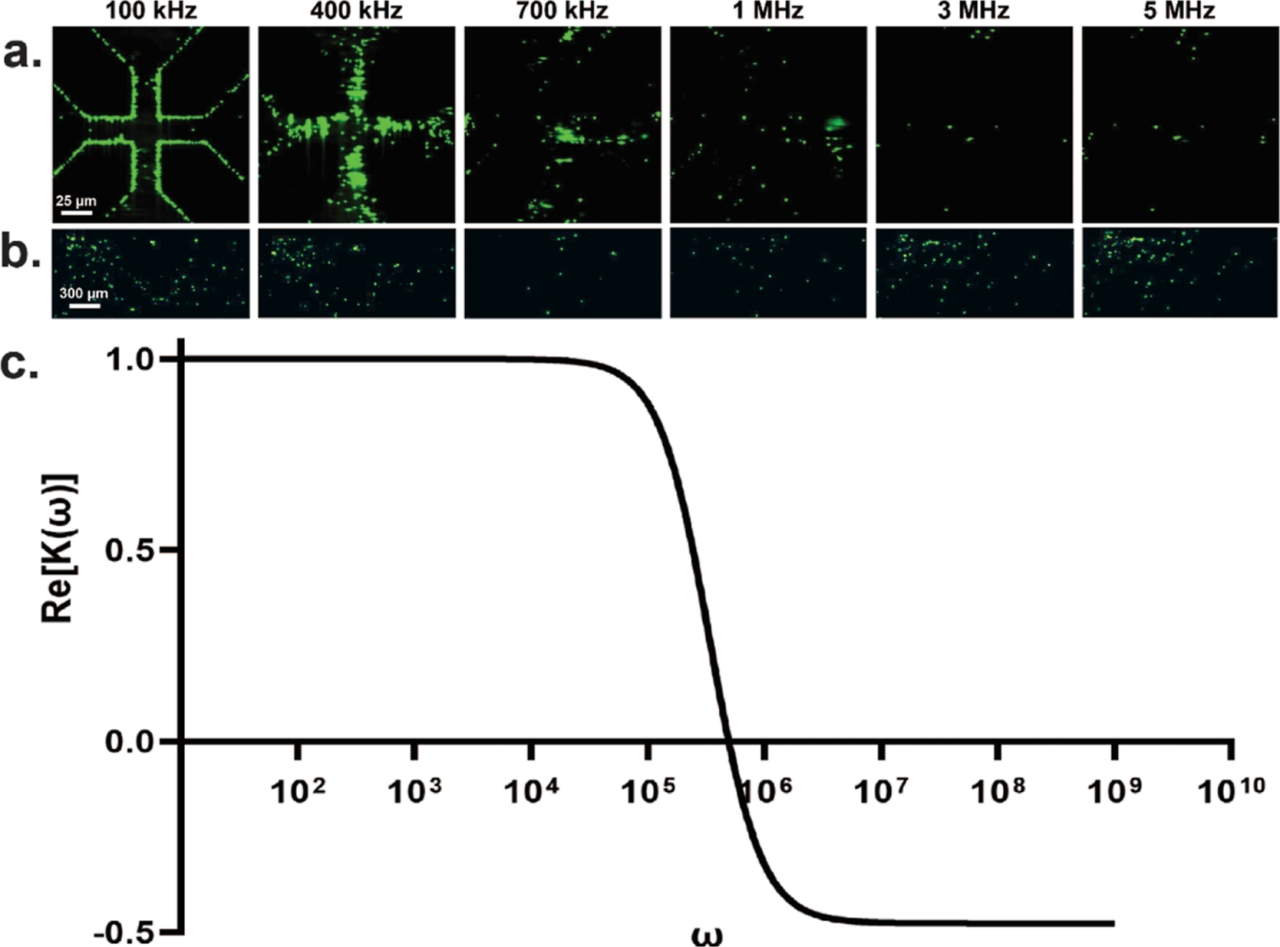
Comparison of PS particle assembly using AC DEP between a traditional 2D quadrupole electrode array (A) and a paper DEP (B). As shown, particle assembly trends are similar in both paper and the quadrupole electrode at low frequency below the COF. At frequency above the COF, we observe significantly greater nDEP trapping in paper. (C) DEP CMF curve generated for 1 μM PS particles depicts a predicted DEP COF at 550 kHz.

As shown in Figure 4a, the DEP crossover frequency (COF) for 1 μm PS particles was determined to be 550 kHz. As anticipated, at frequencies below 550 kHz, we observed the assembly of PS particles under positive DEP (pDEP) near the high-field quadrupole electrode edges (Figure 4a). However, more importantly, we observed particle trapping within the paper pores (Figure 4b). To guide experimental observations, a frequency-dependent CMF curve was computed for 1 μm PS particles using previously published electrical properties (Figure 4c).^48−50^ As shown in Figure 4a,b, as field frequency increases and approaches the COF, particles release from the high field zones—both in paper and on the electrode array—due to particles experiencing a reduced DEP force. Interestingly, we observe particle trapping within paper pores above the DEP COF under negative DEP (nDEP) but observe only minimal particle assembly in the low central electric field region of the quadrupole array. In paper DEP at frequencies above the COF, the particles experience a significant nDEP force but are fluidically confined within their surrounding paper pore. As such, particles become immobilized and trapped at the entrance to these zones of high electric field gradient under nDEP. These results are in agreement with previously reported pillar-type iDEP experiments that showed particles can be trapped under negative DEP when fluidically confined.^51^ We did not observe any temperature increase within the paper DEP device, as measured using an IR thermal imaging thermometer (FLIR TG165). We speculate that the continuous flow of fresh fluid serves to drive any heat by Joule heating away from the DEP trapping zone. Paper-based AC DEP can therefore potentially be used to trap heat sensitive soft matter particulates and biomolecules. These experiments demonstrate that paper-DEP devices show trapping dynamics akin to the traditional electrode-based DEP but at a fraction of the cost of fabrication efforts.

### 4.3 DEP Trapping Using a Pulsed DC Electric Field

**Figure 5:**
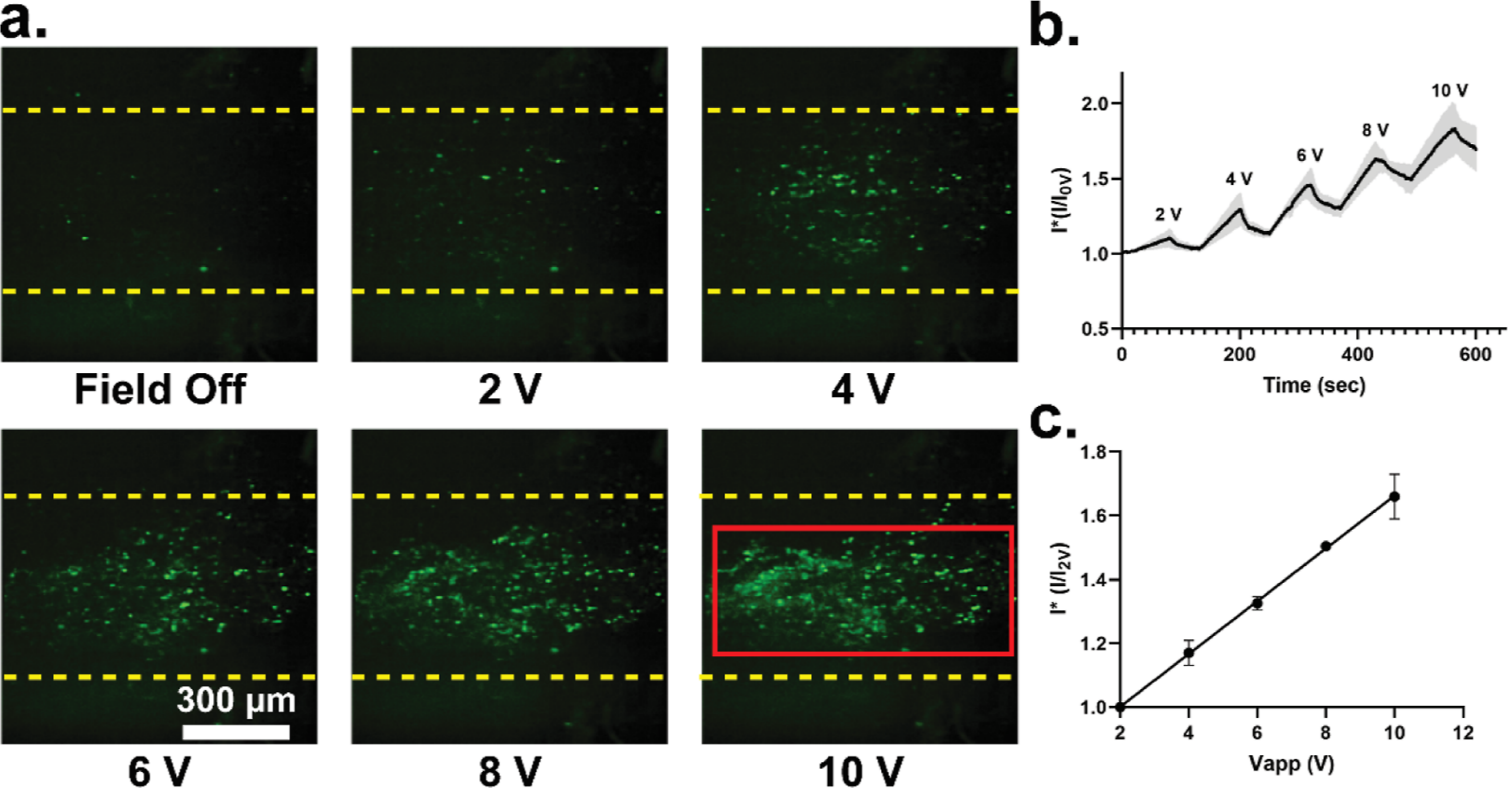
DEP particle trapping of fluorescently labeled PS particles with a PDC field. (A) Micrographs of paper DEP particle trapping at different applied PDC voltages. (B) Particle capture and release dynamics show clear drop in trapped particles when PDC field removed. (C) Maximum fluorescent intensity determined over varying PDC voltages (one standard deviation error bar, *n* = 5 fluorescent measurement for each data point).

To further optimize this method as a low-cost, user-friendly POC platform, we investigated the ability to induce DEP by using a pulsed DC (PDC) electric field. A PS particle suspension was driven through a paper channel at a constant flow rate (4 μL/min), and a PDC field (10 V, 1 kHz, 80% duty cycle) was applied across the copper electrode pair gap. DEP trapping was quantified using confocal microscopy for a given applied electric field strength by capturing fluorescent intensity measurements within given region of interest, as shown by the red boundary in Figure 5a. As shown, the number of particles trapped within the paper pores increases with the DC voltage. Potential reversible trapping-release dynamics were determined by applying a PDC field for 70 s to induce DEP and trap particles, followed by a 50 s removal of the field to release DEP-trapped particles using the flow field. This trap-release process was repeated over a PDC voltage varying from 2–10 V PDC (Figure 5b). For all voltages investigated, when the PDC field was applied, the fluorescence intensity was observed to steadily increase. Subsequently, when the PDC field was removed, the fluorescent intensity was observed to decrease, demonstrating that trapping of PS particles within the paper occurs solely due to the applied PDC field. It is important to note that perfect hysteresis of particle trapping-release was not observed, and there was a degree of irreversible particle retention occurring within the paper pores. While the BSA treatment alleviated irreversible particle adsorption, investigation into surfactant and other paper passivation techniques will be a subject of future work to better optimize this platform. The peak fluorescent intensity as measured for each PDC voltage is shown in Figure 5c. As depicted, particle trapping is observed to scale linearly with the applied PDC voltage and agrees well with the trapping dynamics predicted by our FEA calculations (Figure 3).

### 4.4 Paper DEP Comparison between Constant Flow Rate and Capillary Flow

**Figure 6:**
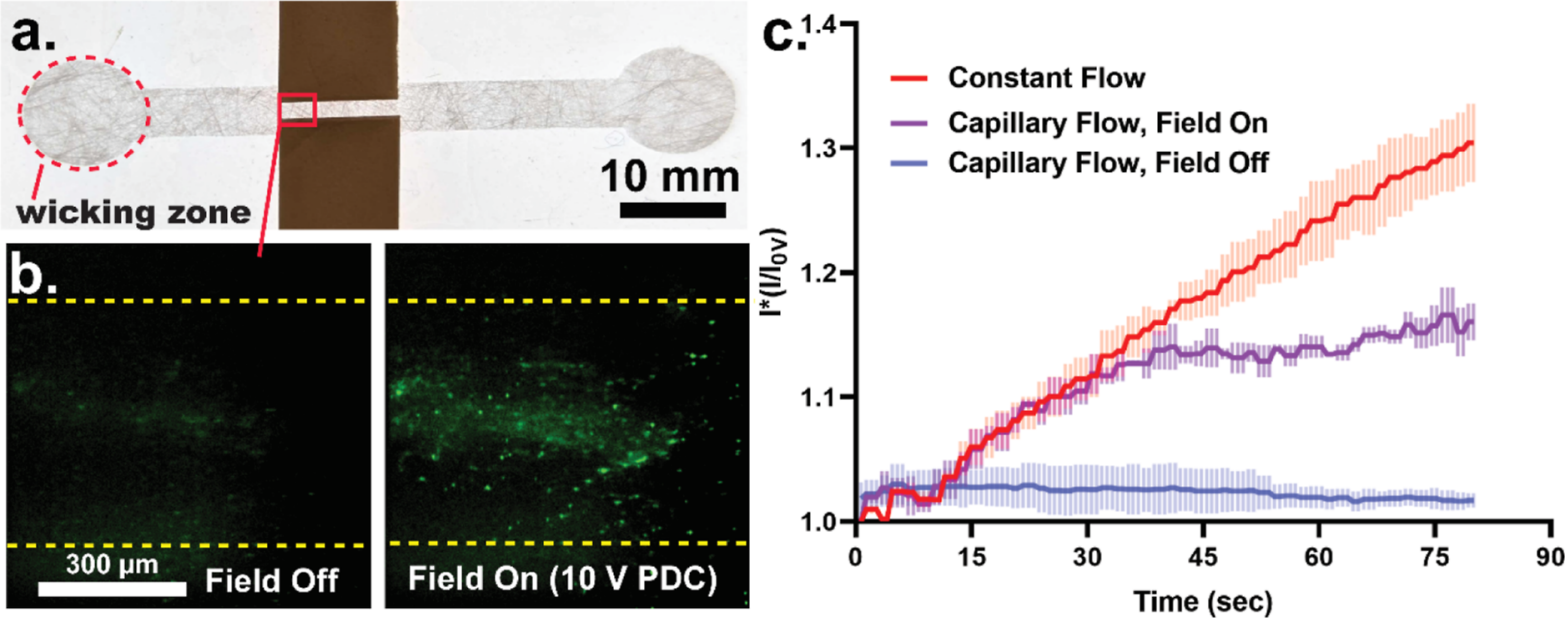
DEP particle trapping under passive wicking traps fewer particles than that under a constant pressurized flow. (A) DEP PS particle trapping with field off and on (10 V) under capillary flow. (B) Comparison of DEP trapping between constant fluid flow and capillary wicking (one standard deviation error bar for *n* = 3 measurements).

One advantage of paper-based devices is the ability to drive fluid flow by capillary action. We investigated paper DEP trapping in paper under capillary action and compared the performance to devices driven with an external driven flow. First, a 50 μL of PS solution was pipetted onto a circular nonlaminated paper wicking zone (Figure 6a). The fluid was wicked and started flowing through the paper channel. When the fluid front wicked into and passed the electrode region, we applied a 10 V PDC electric field and measured the PS-induced fluorescent intensity. This experiment was repeated in the absence of an electric field (Figure 6a), and the DEP trapping performance was compared to that under active fluid flow through a μPiP channel with a constant flow rate of 4 μL/min over a time period of 80 s. Under a sustained μPiP flow, there is a constant convective flux of particles entering the DEP trapping zone. As shown in Figure 6b, trapping-induced fluorescent intensity, driven with constant flow rate, increases linearly with time. However, for trapping under capillary flow, excluding surface evaporation, the flow rate can be approximated using the Lucas–Washburn equation, where fluid flow ∼ t^1/2^.^28^ As expected, the flux of particles entering the trapping zone decreases with increasing time and approaches a plateau, which is conveyed by the observed fluorescence intensity DEP trapping data. Furthermore, no trapping is observed under capillary flow in the absence of a PDC field (Figure 6b). Therefore, particles are trapped solely by DEP; gravitational and mechanical trapping do not play any role in our paper-based fluidic system.

### 4.5 Dielectrophoretic Trapping of *E. coli* in Paper

**Figure 7:**
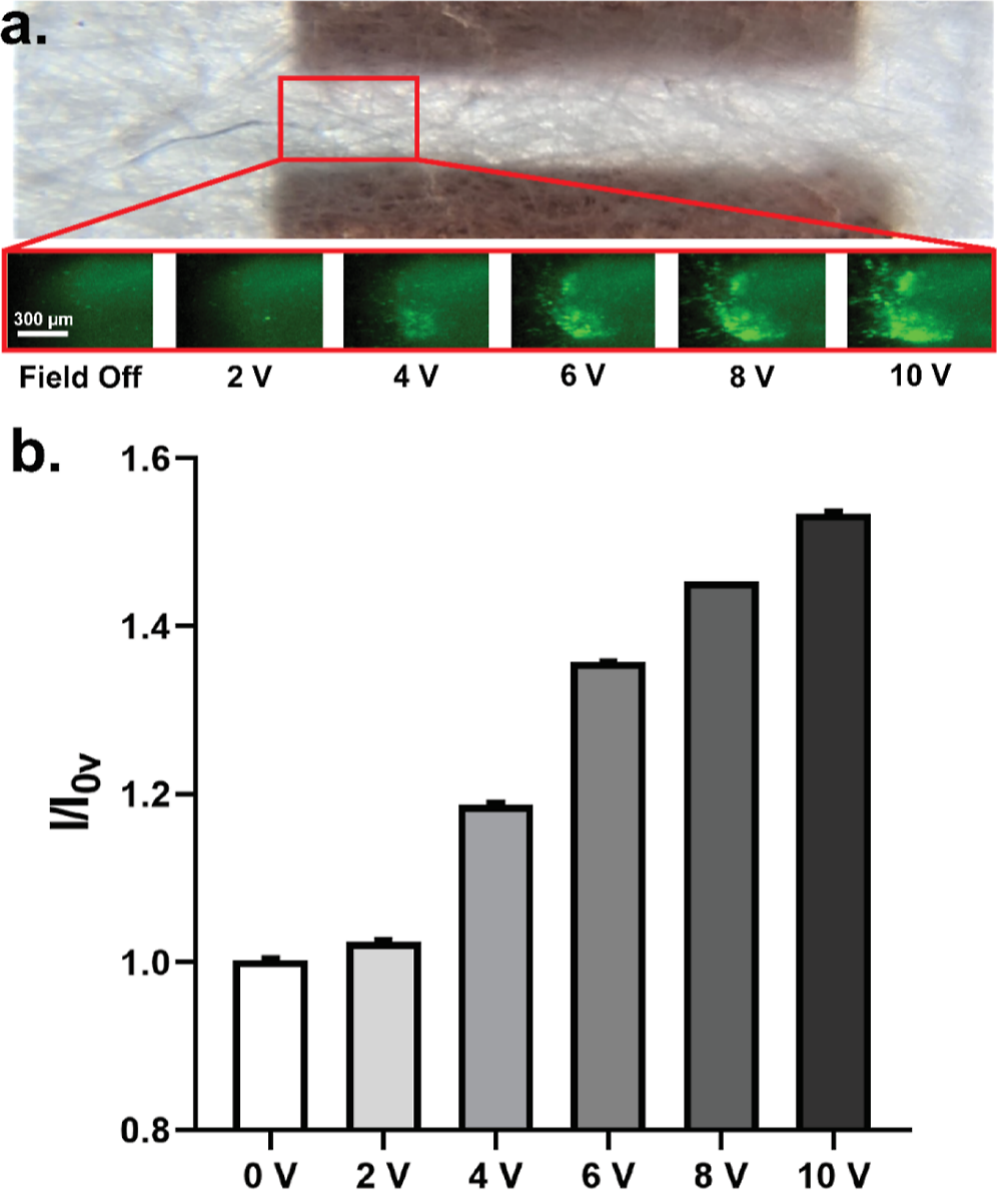
Paper-DEP trapping of *E. coli* bacteria. (A) Cell trapping, measured in the boxed region of interest, obtained via fluorescent intensity measurement over varying PDC voltages (0–10 V PDC). (B) Fluorescent intensity captured at different PDC voltages after 2 min of paper DEP trapping (one standard deviation error bar for *n* = 5 measurements).

With the ability to trap PS particles using paper DEP, we next sought to demonstrate the biocompatibility of this method by dielectrophoretically trapping *E. coli* cells in the paper pores. A suspension of SYBR-treated *E. coli* cells was flowed through paper at a constant flow rate of 4 μL/min, and a PDC field was applied across the paper channel for a duration of 2 min at varying voltages ranging between 0 and 10 V PDC. The resulting fluorescent intensity was measured for each voltage within a given region of interest, as depicted in the red barrier shown in Figure 7a. Furthermore, we observe a nonuniform cell trapping distribution because cells are efficiently trapped by DEP as soon as they enter the electric field zone. Within this porous DEP trapping region, the measured fluorescent intensity is observed to increase with the applied voltage (Figure 7b).

## 5 Conclusions

In this work, we have demonstrated a novel paper-based DEP mechanism utilizing low-cost commercially available insulating paper fibers. The microscale porous nature of this material can generate localized zones of high electric field gradient and a subsequent DEP force within the paper structure similar to conventional iDEP devices. These paper DEP trapping zones can be used to trap and concentrate particles as well as living cells. Using a combination of micro-CT imaging and FEA, we quantitatively calculated and predicted the pore-scale DEP force growth dynamics and trapping mechanism within these paper pores. Using this low-cost substrate, we demonstrated particle trapping with AC-based DEP and compared paper DEP trapping with a conventional 2D quadrupole electrode array. At low frequency below the COF PS particles were observed to be trapped under pDEP in both the paper pores and the electrode edges. However, at high frequency above the COF, the particles were observed to trap under nDEP. To further simplify device cost for POC applications, we then demonstrated paper DEP by using a PDC electric field and observed a linear relationship between the DEP trapping concentration and the PDC voltage. This experiment was repeated using two different fluid flow mechanisms—a constant μPiP-type flow and passive capillary wicking—demonstrating that paper DEP can also be successfully performed using a passive flow. Finally, we utilized this method to successfully capture *E. coli* bacteria in the paper pores. Unlike conventional iDEP devices, fabrication is rapid and low-cost and does not require a cleanroom. We believe that this novel paper-DEP method has immediate and broad applications, including sample preparation, disease diagnostics, microbial contamination detection, public health monitoring, and synthetic biology. With further development, this technique will democratize DEP-based innovations by significantly reducing fabrication costs and enable the manufacturing of robust devices at a commercial scale.
